# Association of Epstein-Barr virus infection with peripheral immune parameters and clinical outcome in advanced nasopharyngeal carcinoma

**DOI:** 10.1038/s41598-020-78892-0

**Published:** 2020-12-15

**Authors:** Dan Tao, Ningning Zhang, Qingqing Huang, Chuang Ge, Qicheng Li, Shujie Li, Kegui Weng, Qishuai Guo, Jiangdong Sui, Can Wang, Xin Zhang, Ying Wang

**Affiliations:** 1 Department of Radiation Oncology, Chongqing University Cancer Hospital & Chongqing Cancer Institute & Chongqing Cancer Hospital, Chongqing, China; 2 Chongqing Key Laboratory of Translational Research for Cancer Metastasis and Individualized Treatment, Chongqing University Cancer Hospital, Chongqing, China; 3 Breast Cancer Center, Chongqing University Cancer Hospital & Chongqing Cancer Institute & Chongqing Cancer Hospital, Chongqing, China; 4 Department of Nutrition, Chongqing University Cancer Hospital & Chongqing Cancer Institute & Chongqing Cancer Hospital, Chongqing, China; 5 Department of Clinical Laboratory, Chongqing University Cancer Hospital & Chongqing Cancer Institute & Chongqing Cancer Hospital, Chongqing, China

**Keywords:** Cancer therapy, Head and neck cancer, Oncology

## Abstract

The purpose of this study was to investigate the association of Epstein-Barr virus (EBV) with peripheral blood immune cell counts and clinical outcomes in advanced nasopharyngeal carcinoma (NPC) patients. In a retrospective design, 146 patients with NPC at stage IV were enrolled in this study. The association of EBV status with peripheral blood immune cell counts, distant metastases, and long-term survival in patients with advanced NPC were determined. Eighty-seven (59.6%) of all patients were positive for EBV. Compared with patients with normal NK cell count, patients with lower NK cell count showed a significantly lower EBV viral load (median: 614.0 *vs.* 2190.0 copies/mL,* P* = 0.024). EBV-positive patients showed a significantly higher incidence of liver metastasis than EBV-negative patients (32.6% vs. 23.7%, *P* = 0.021). Multi-variant regression analysis showed that EBV infection was independently associated with liver metastasis (OR: 2.33, *P* = 0.043). EBV positive patients showed a significantly worse PFS (*P* = 0.001) and OS (*P* = 0.001) than EBV negative patients. Multivariate Cox regression analysis revealed that EBV infection was independently associated with a worse PFS (HR: 1.94, *P* = 0.003), and OS (HR: 2.12, *P* = 0.014) in advanced NPC. In conclusion, EBV infection is associated with a high risk of liver metastasis and is also an independent negative predictor for PFS and OS in patients with advanced NPC. EBV infection is associated with lower CD8% and higher NK%, while lower NK cell count is associated with lower EBV viral load.

## Introduction

Nasopharyngeal carcinoma (NPC) occurs worldwide and is endemic in southern China and Southeast Asia^1^. More than 90% of NPC are poorly differentiated squamous cell carcinoma and undifferentiated carcinoma^2^. Thus, NPC is relatively sensitive to radiotherapy and chemotherapy^1^. More than 80% of early-stage NPC is curable with radiation therapy and chemotherapy^3^. However, due to the biological characteristics of highly invasive and metastatic potential, advanced stage NPC with either locally and metastatic at onset or recurrent has an unfavorable prognosis. Hence, studies to explore factors influencing the process of pathogenesis and development, and the therapeutic effects of NPC are warranted.

NPC is closely associated with the Epstein-Barr virus (EBV). EBV plays an essential role in the initiation and progression of NPC^4,5^. The plasma EBV DNA load is a highly sensitive and specific tumor marker in NPC patients^6–8^. Plasma EBV DNA load is significantly correlated with tumor burden and associated with disease stage and prognosis^8–13^. However, not all advanced NPC patients have a higher EBV viral load, and the EBV viral load is constantly changing in the progression of the tumor. Thus, studies are needed to find the susceptibility factor of EBV replication and special point in the promotion of metastasis by EBV in advanced NPC.

EBV causes asymptomatic lifelong infection in nearly all humans by adulthood. Both host humoral and cellular immune responses can control but not eliminate the proliferation of EBV-infected cells in healthy virus carriers. During the primary EBV infection, both T cell-mediated EBV-specific responses and NK cell-mediated nonspecific responses play an essential role^14^. During latent infection, T cells played a pivotal role in inhibiting the proliferation of EBV-infected cells^15,16^. When the immune system of the body is in a disordered status, persistent EBV infection can lead to the development of EBV-positive malignant tumors, such as NPC. It is well known that immune-suppression contributes to the initiation and progression of NPC and the immune function is correlated with the prognosis of NPC patients^17,18^. However, the association of EBV infection status with peripheral immune parameters and clinical outcomes in patients with advanced NPC is still not clear.

In this study, we examined the interaction between EBV infection and peripheral blood immune cells and its relationship with clinical outcome, aiming to elucidate the clinical role of EBV and factors affecting clinical outcome in advanced NPC and to provide insights to the treatment of advanced NPC.

## Material and methods

### Patients and data collection

This study was approved by the Medical Ethics Committee of the Chongqing University Cancer Hospital. For this retrospective study, the Institutional Ethics Committee waived the need for informed consent. All methods were performed in accordance with the relevant guidelines and regulations.

The patients with histologically confirmed nasopharyngeal carcinomas (NPC) were screened from Chongqing University Cancer Hospital (Chongqing, China) between January 2011 and November 2018. Histopathology was based on the WHO international histological classification. All tumors were staged according to the American Joint Committee on Cancer classification. Eligible patients were those who were ≥ 18 years; staged IV; had complete information on EBV status and immune function variables. For all patients, we reviewed the clinical records to obtain clinicopathologic characteristics, including age, sex, smoking history, tumor histology, and the information of metastasis and treatments.

### EBV detection

Plasma samples of patients were prepared from the peripheral blood by centrifugation at 3000 rpm for 10 min and then stored at -80 °C until use. The real-time quantitative PCR was used to detect EBV-DNA. According to the manufacturer (ZJ Bio-Tech Co., Ltd., Shanghai, China), it was defined as positive if EBV-DNA were higher or equal to 500 copies/mL in plasma. All the experiments were conducted as per the manufacturer's instructions.

### Blood samples and flow cytometry for the detection of immune function

The percentage and the absolute number of CD3+, CD4+, CD8+, CD16+ CD56+ , and CD19+ lymphocytes were detected by FACS Calibur cytometer (BD Biosciences) as follows: peripheral blood specimens drawn by venipuncture were collected by using BD Vacutainer EDTA Tubes (K3 EDTA). The samples were then analyzed using Facs lysing solution according to the manufacturer's instructions (BD MultiTest IMK kit). The kit comprised of a panel of monoclonal antibodies of fluorescein isothiocyanate (FITC)-labeled CD3; CD8 phycoerythrin (PE); CD45 peridinin chlorophyll protein (PerCP); CD4 allophycocyanin (APC); CD16+ 56-PE; and CD19-APC. Briefly, 50 μL of whole blood (EDTA) was added to 20 μL of both fluorochrome-conjugated monoclonal antibodies (anti-CD3 FITC/CD8 PE/CD45 PerCP/CD4 APC and anti-CD3 FITC/CD(16 + 56) PE/CD45 PerCP/CD19 APC. Tubes were vortexed and incubated for 15 min in the dark at room temperature. Four hundred fifty microliters of 1× MultiTest lysing solution was added to each tube, and tubes were vortexed and incubated for 15 min at room temperature in the dark. At last, the specimens were then analyzed by FACSCalibur cytometer. Data acquisition and analysis were performed by computerized calculations using Cell Quest software^19^.

The normal value of peripheral immune parameters were defined according to the manufacture’s recommendations as follows: percentage of total T lymphocyte (CD3%): (50–84)%; total T lymphocyte (CD3) count: (955–2860) × 10^6^/L; percentage of inhibition of T lymphocyte (CD8%): (15–44)%; inhibition of T lymphocyte (CD8) count: (320–1250) × 10^6^/L; percentage of helper T lymphocyte (CD4%): (27–51)%; helper T lymphocyte (CD4) count: (550–1440) × 10^6^/L; the ratio of CD4 and CD8 (CD4/CD8): 0.71–2.87; percentage of natural killer cell (NK%): (7–40)%; natural killer cell (NK) count: (150–1100) × 10^6^/L; percentage of B lymphocyte (BC%): (5–18)%. B lymphocyte (BC) count: (90–560) × 10^6^/L; total lymphocyte count (CD45): (1530–3700) × 10^6^/L.

### Statistical analysis

The associations between EBV status and immune function variables and clinicopathologic variables were evaluated by Kruskal–Wallis tests (for continuous variables) or Pearson chi-square test (for categorical variables), as appropriate. PFS was defined as the time interval from the date of diagnosis of stage IV disease to the date of disease progression or death. OS was defined as the time interval from the date of diagnosis of stage IV disease to the date of death. PFS and OS were calculated by the Kaplan–Meier method. The Log-rank test was used for intergroup comparisons. Cox proportional hazards model was used to perform univariate and multivariate analyses of PFS and OS. Logistic regression was used for univariate and multivariate analysis of binary variables. Baseline variables that were considered clinically relevant or that showed a significant relationship in univariate analysis were entered into multivariate regression. A *P* value of < 0.05 was taken as being statistically significant. R (version 3.6.2; http://www.r-project.org ) software was used to analyze data.

## Results

### Patients characteristics

In total, 146 patients were enrolled in this study. Of these patients, 113 (77.4%) were male and 33 (22.6%) were female, with age ranging from 21 to 78 years (median 50.07 years). Eighteen patients were stage IVa, 22 patients were stage IVb, and 106 patients were stage IVc. Eighty-seven of all patients showed EBV positive. NPC patients with EBV positive were significantly associated with liver metastasis (*P* = 0.040), and no significant difference was observed between EBV positive with other clinicopathological parameters. The baseline clinical characteristics of these enrolled patients and the association between EBV infection and clinicopathological features are shown in Table 1.

**Table 1:** Characteristics of patients with advanced nasopharyngeal carcinoma according to EBV status.

Variables	Total patients N = 146 (%)	EBV infection		
		Positive N = 87 (%)	Negative N = 59 (%)	P value
**Age at IV stage, yrs**				
Mean SD (median)	50.07 ± 11.25	50.11 ± 11.02	50.00 ± 11.77	0.952
Range	21–78	26–75	21–78	
< 50	73 (50.0)	44 (50.6)	29 (49.2)	0.866
≥ 50	73 (50.0)	43 (49.4)	30 (50.8)	
**Sex**				0.060
Male	113 (77.4)	72 (82.8)	41 (69.5)	
Female	33 (22.6)	15 (17.2)	18 (30.5)	
**Smoker**				0.521
Yes	74 (50.7)	46 (52.9)	28 (47.5)	
No	72 (49.3)	41 (47.1)	31 (52.5)	
**HBsAg**				0.403
Positive	21(15.3)	14(17.5)	7(12.3)	
Negative	116(84.7)	66(82.5)	50(87.7)	
**T-stage**				0.871
Tx-3	105 (71.9)	63 (72.4)	42 (71.2)	
T4	41 (28.1)	24 (27.6)	17 (28.8)	
**N-stage**				0.370
N0-2	102 (69.9)	58 (19.0)	44 (74.6)	
N3	44 (30.1)	29 (81.0)	15 (25.4)	
**M-stage**				0.147
M0	40 (27.4)	20(23.0)	20 (33.9)	
M1	106 (72.6)	67 (77.0)	39 (66.1)	
**Clinical stage**				0.349
IVa	18 (12.3)	9 (10.3)	9 (15.3)	
IVb	22 (15.1)	11 (12.7)	11 (18.6)	
IVc	106 (72.6)	67 (77.0)	39 (66.1	
**Lung metastases**				0.789
Yes	32 (21.9)	19 (21.8)	13 (22.0)	
No	114 (78.1)	68 (78.2)	46 (78.0)	
**Liver metastases**				**0.040**
Yes	38 (26.0)	28 (32.2)	10 (16.9)	
No	108 (74.0)	59 (67.8)	49 (83.1)	
**Bone metastases**				0.121
Yes	53 (36.3)	36 (41.4)	17 (28.8)	
No	93 (63.7)	51 (58.6)	42 (71.2)	
**Prior radiotherapy**				0.278
Yes	59 (40.4)	32 (36.8)	27 (45.8)	
No	87 (59.6)	55 (63.2)	32 (54.2)	
**Prior chemotherapy**				0.220
Yes	58 (39.7)	31 (35.6)	27 (45.8)	
No	88 (60.3)	56 (64.4)	32 (54.2)	
**HBsAg positive**				0.345
Yes	21 (14.4)	14 (16.1)	7 (11.9)	
No	116 (79.4)	66 (75.9)	50 (84.7)	
Unknown	9 (6.2)	7 (8.0)	2 (3.4)	

### Relation of peripheral immune parameters and clinical features to EBV infection

**Figure 1:**
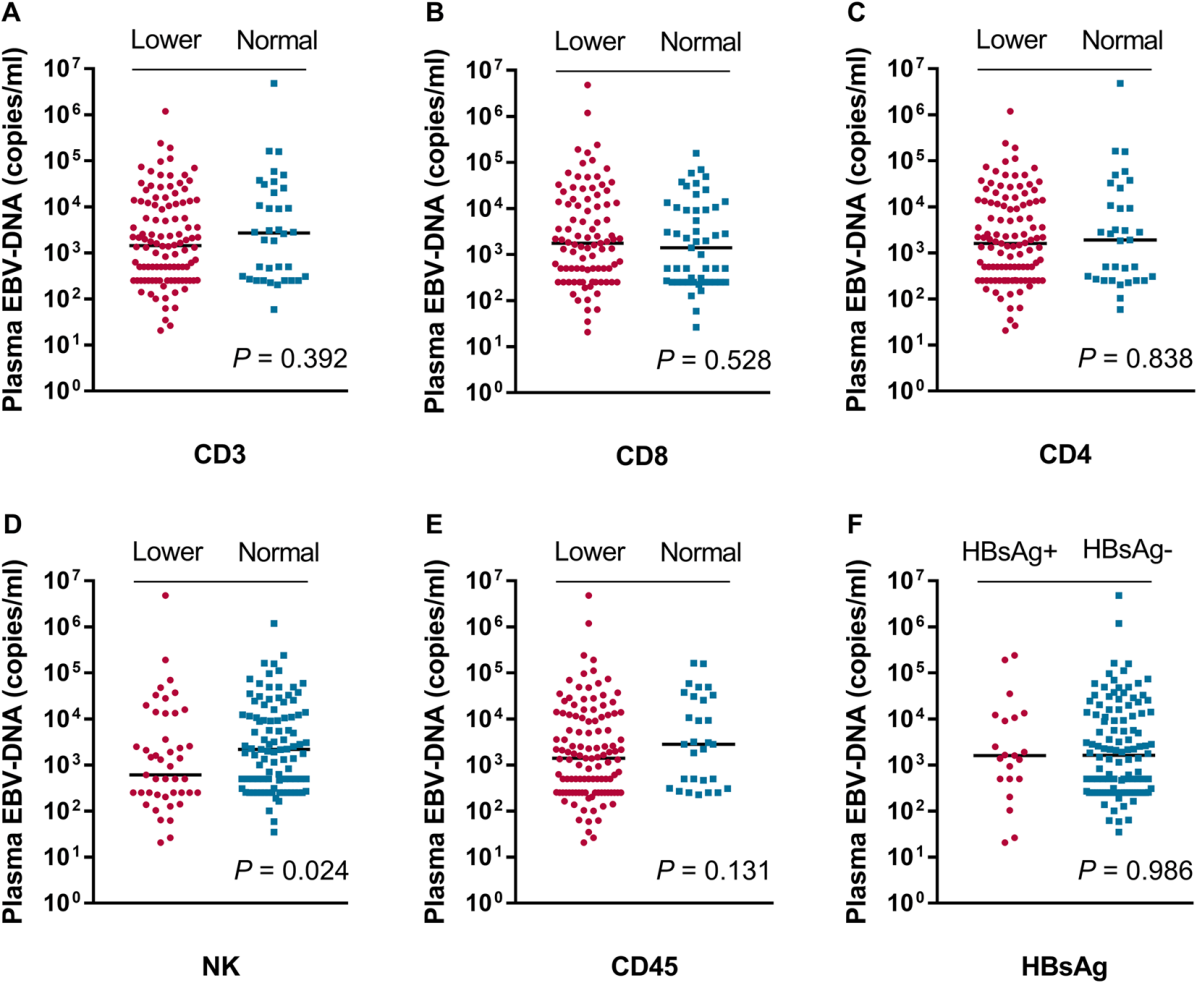
Comparison of EBV DNA load in patients with NPC between the different status of the immunity parameter (**A**–**E**) and HBsAg (**F**). Lower: indicate that the value is lower than the normal range; Normal: indicates that the value is in the normal range or higher than the normal range.

As displayed in Supplementary Table 1, EBV positive status was significantly related to CD8% (*P* = 0.042) and NK% (*P* = 0.040), but no significant association with other immune indexes. It is well known that the EBV-DNA load is a surrogate for tumor burden in NPC patients. In this study, we further evaluated the relation of EBV viral load to clinical characterization and peripheral immune parameters. As shown in Fig. 1, the EBV viral load of patients with lower NK cell count was significantly associated with lower EBV viral load (median: 614.0 *vs.* 2190.0 copies/mL, *P* = 0.024; Fig. 1D). EBV viral load did not show a significant relationship with other immunity parameters.

### Impact of EBV infection on the incidence and sites of metastasis

**Figure 2:**
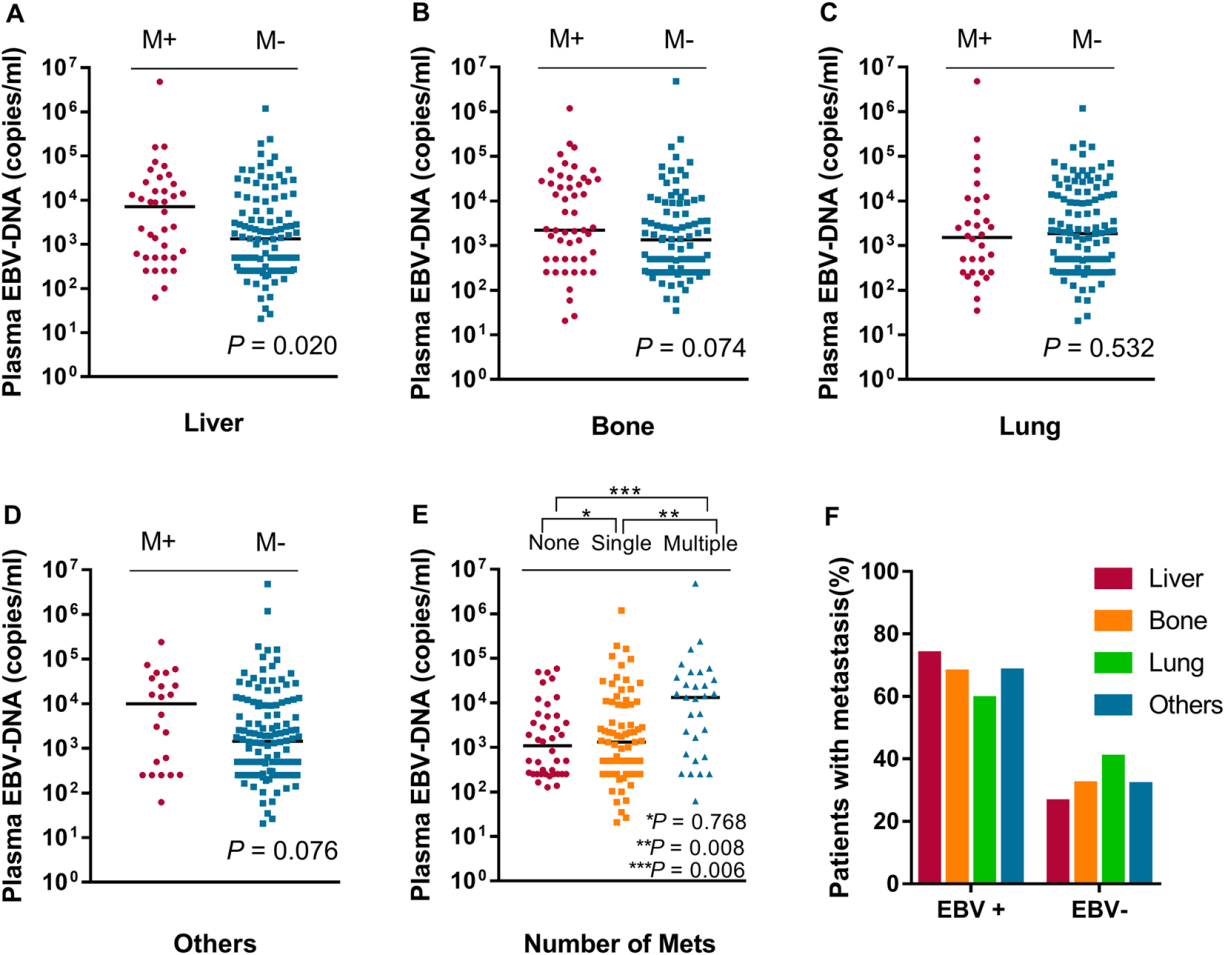
Comparison of EBV DNA load in patients with NPC according to different metastatic sites and the number of organ metastases. (**A**–**D**) Plasma EBV DNA levels of patients with NPC with or without liver (**A**), bone (**B**), lung (**C**), and other organ metastasis (**D**). (**E**) Plasma EBV DNA levels of patients with NPC according to the number of metastasis. (**F**) Pattern of organs metastasis according to the different EBV infection status. M, metastasis.

One hundred and twenty-six (75.9%) patients had distant metastasis in this cohort study. Of these patients, 38 (22.9%), 32 (19.3%), 53 (31.9%), and 22 (13%) had liver, lung, bone, and other metastasis, respectively. The rate of distant metastasis in EBV positive patient (77%, 67/87) was higher than that in EBV negative patient (66.1%, 39/59). However, the difference in the rate of distant metastasis between EBV positive patient and negative patients did not achieve a significant level (*P* = 0.147). Patients with liver metastasis showed significantly higher EBV DNA load than patients without liver metastasis (7125.0 vs 1335.0 copies/mL, *P* = 0.020; Fig. 2A). Patients with bone or other metastasis also showed a higher EBV DNA load than those without bone or other metastasis (including non-regional lymph node, adrenal gland, etc.) (bone: 2230.0 vs 1350.0 copies/mL, *P* = 0.074; others: 9905.0 vs 1445.0 copies/mL, *P* = 0.076; Fig. 2B, 2D), but the differences in the EBV load between two groups were not statistically significant. The difference in the EBV load between patients with and without lung metastasis did not achieve a significant level (1525.0 vs 1870.0 copies/mL, *P* = 0.532; Fig. 2C).

To investigate whether there is indeed an association between EBV infection and liver metastasis in advanced NPC patients, univariate and multivariate logistic regression analysis was performed. The univariate logistic analysis showed that EBV infection was significantly associated with liver metastasis (OR: 2.325, 95%CI: 1.029–5.256, *P* = 0.043; Table 2). Multiple logistic analyses demonstrated that EBV infection was a significant and independent risk predictor for liver metastasis in NPC patients (OR: 2.856; 95%CI: 1.193–6.837, *P* = 0.018). However, unexpectedly, the results showed that the T4 stage was an independent protective factor for liver metastasis (OR: 0.600; 95%CI: 0.436–0.825, *P* = 0.002). No significant effect of immune parameters was found on liver metastasis.

**Table 2:** Univariate and multivariate analyses of various predictive factors for liver metastasis in patients with advanced nasopharyngeal carcinoma.

Variables	Univariate analysis OR (95% CI)	*P* value	Multivariate analysis OR (95% CI)	*P* value
**Sex**		0.525		
Male	Reference			
Female	1.320 (0.560–3.108)			
**Age**		0.304		
< 65	Reference			
≥ 65	0.444 (0.095–2.084)			
**Smoker**		0.922		
No	Reference			
Yes	0.964 (0.460–2.078)			
**T-stage**		**0.004**		**0.002**
Tx-3	Reference		Reference	
T4	0.630 (0.462–0.860)		0.600 (0.436–0.825)	
**N-stage**		0.160		0.060
N0-2	Reference		Reference	
N3	0.533 (0.222–1.281)		0.406 (0.158–1.041)	
**HBsAg**		0.253		0.641
No	Reference		Reference	
Yes	1.764 (0.666–4.672)		1.284(0.449–3.670)	
**EBV**		**0.043**		**0.018**
No	Reference		Reference	
Yes	2.325(1.029–5.256)		2.856(1.193–6.837)	
**CD3**		0.550		
No	Reference			
Yes	1.312(0.539–3.199)			
**CD8**		0.288		
No	Reference			
Yes	1.534(0.697–3.376)			
**CD4**		0.475		
No	Reference			
Yes	1.404(0.553–3.563)			
**NK**		0.925		
No	Reference			
Yes	0.963(0.435–2.129)			
**CD45**		0.796		
No	Reference			
Yes	0.133(0.441–2.913)			

### Impact of EBV infection on the pattern of distant metastasis

Of 146 enrolled patients, distant metastasis occurred in 126 patients, 0, 1 (single) and ≥ 2 (multiple) sites accounted for 24.1%, 45.2% and 18.7%, respectively. Patients with multiple metastasizes showed a significantly higher EBV DNA load than those with single or without metastasis (multiple *vs.* single: 13,300.0 *vs.* 1320.0 copies/mL, *P* = 0.008; multiple *vs.* none: 13,300.0 vs 1093.0 copies/mL, *P* = 0.006; Fig. 2E). However, the difference in EBV DNA load between patients with single and without metastasis was not significant (single *vs.* none: 1320.0 vs 1093.0 copies/mL, *P* = 0.768). In patients with liver, bone, lung, and other metastasis, 73.7% (28/38), 67.9%(36/53), 59.4%(19/32), and 68.2% (15/22) showed EBV positive, respectively (Fig. 2F).

### Association of HBsAg status with EBV infection and liver metastasis

A total of 137 patients with HBV status was analyzed. Of these patients, 12.7% (21/137) were HBsAg positive. Of these HBsAg positive patients, 66.7% (14/21) was EBV positive, which was higher than those HBsAg negative patients (33.3%, 7/21). However, no significant difference was observed between the two groups (*P* = 0.403). The EBV viral load of patients with HBsAg positive status was similar to that of patients with HBsAg negative status (median: 1610.0 vs. 1620.0, *P* = 0.986; Fig. 1F). Some previous studies showed that HBV infection was associated with liver metastasis. Our results demonstrated that HBsAg positive patients showed a higher rate of liver metastasis than HBsAg negative patients (38.1% vs 25.9%), but this difference was not achieved statistical significance (*P* = 0.249). Neither univariate nor multivariate logistic regression analysis (univariate analysis OR: 1.764, 95%CI: 0.666–4.672, *P* = 0.253; multivariate analysis OR: 1.284, 95%CI: 0.449–3.670, *P* = 0.641; Table 2) showed a significant effect of HBsAg positive status on liver metastasis.

### Association of EBV infection and immunity parameters with PFS and OS

**Figure 3:**
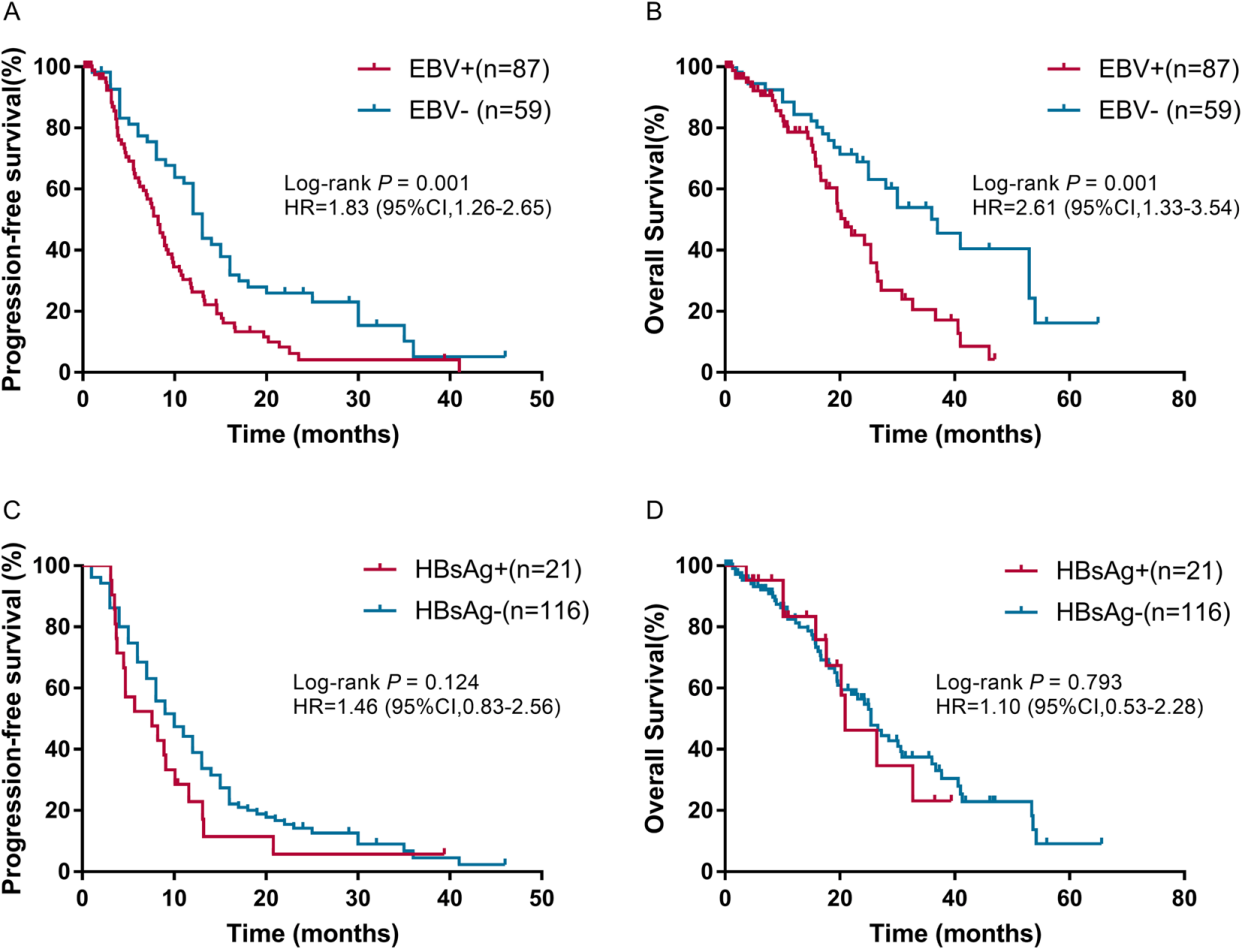
Kaplan–Meier estimate of PFS and OS according to the EBV (**A**,**B**) and HBsAg status (**C**,**D**). PFS, progression-free survival; OS, overall survival.

**Figure 4:**
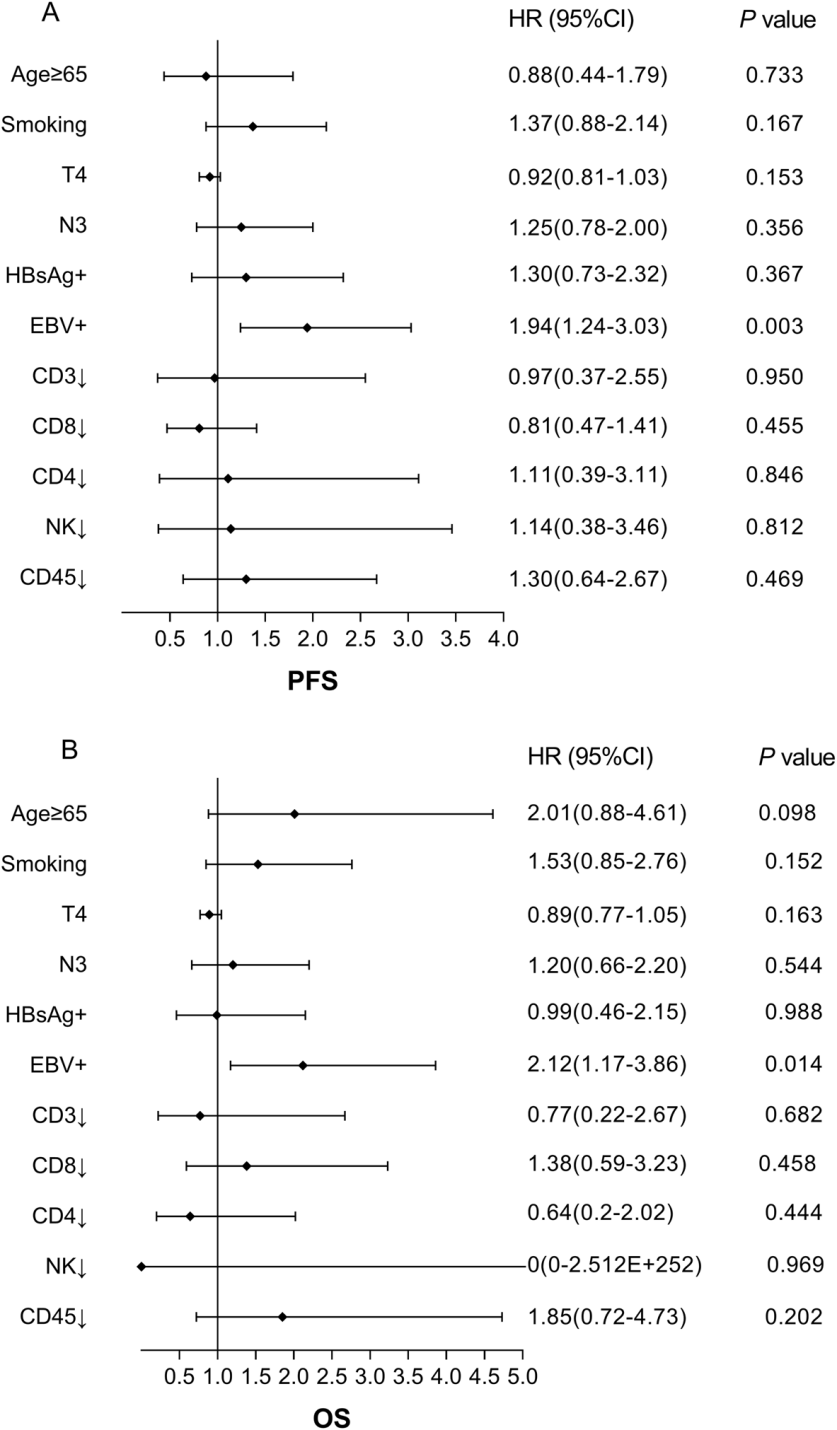
Multivariate analysis of variables correlated with PFS (**A**) and OS (**B**). PFS, progression-free survival; OS, overall survival.

Kaplan–Meier survival analysis and log-rank test revealed that EBV infection was significantly associated with worse PFS (mPFS: 8.2 vs. 13.0 months; *P* = 0.001, Fig. 3A) and OS ( mOS: 20.9 vs. 30 months; *P* = 0.001, Fig. 3B). No significant association was observed between the HBsAg status (Fig. 3C, 3D) and immunity parameters (data not shown) and PFS and OS. All variables significant on univariate analysis and those potential prognostic impact factors were entered into the Cox proportional hazard model for the prediction of survival. Cox's regression analysis demonstrated that EBV positive status was a significant and independent worse predictor of PFS (HR: 1.94; 95%CI: 1.24–3.03; *P* = 0.003; Fig. 4A) and OS (HR: 2.12; 95%CI: 1.17–3.86; *P* = 0.014; Fig. 4B) in advanced NPC.

## Discussion

Our study confirmed that EBV infection would raise the risk of liver metastasis in patients with NPC. Logistic analysis showed that EBV infection was an independent risk factor for liver metastasis in NPC patients. However, no significant effect of HBsAg positive status on liver metastasis was found in patients with NPC. EBV positive status was an independent worse prognostic factor affecting PFS and OS. To the best of our knowledge, this is the first study to evaluate the association of EBV infection with immunity parameters. The patients with lower NK cell count was significantly associated with lower EBV viral load. EBV viral load did not show a significant relationship with other immunity parameters.

Previous studies reported that EBV infection was associated with distant metastases in NPC patients^20^, and the plasma EBV DNA load of patients with liver metastasis was higher than that of patients with lung or bone metastasis^8,21^. Consistent with previous findings, we also found that EBV DNA levels of patients harboring live metastasis were higher than these patients harboring bone or lung metastasis in advanced NPC. And metastatic NPC patients with liver metastasis had a higher rate of EBV positive status. This phenomenon can be explained by irregularly expressed cytokines in EBV positive NPC. Previous studies revealed that the EBV DNA load is correlated with IP-10, IL-8, IL-6, and MIP-3α. These cytokines showed a close relationship with liver metastasis. Some studies^22,23^ have shown that elevated serum IP-10 levels were significantly associated with liver metastasis. However, it is not well known how EBV affects these cytokines. The detailed molecular mechanism between EBV infection and liver metastasis is worthy to further investigate.

HBsAg carriers accounted for approximately 10–12% of the general population in the endemic area. In our study, the rate of HBsAg seropositivity in this cohort of patients was 12.7%, generally consistent with the previous report (10.9%)^24^. Xu’s study^25^ demonstrated that HBV infection was significantly associated with the presence of liver metastasis. It is said that HBV infection could cause autoimmune reactions to injury the liver cells directly and establish an immune microenvironment that is suitable for metastasizing tumor cells. However, in our study, we failed to confirm that HBsAg positive status was a significant risk factor for liver metastasis in univariate and multivariate logistic regression analysis. The effect of HBV infection on liver metastasis is still a controversial issue. Interestingly, the phenomenon differs depending on the cancer types with HBV infection, raising the risk of liver metastasis in NPC^25^ and pancreatic cancer^26^, but decreasing in colorectal cancer^27^.

Immune dysfunction promotes the pathogenesis of EBV and contributes to the development of NPC ^3,28^. Some scholars believe that EBV infection should be considered an EBV-associated T/NK-lymphoproliferative disorder rather than a simple form of EBV infection. Previous studies also reported that NK cells, B and T lymphocytes play important roles not only during EBV infection but also in the pathogenesis EBV-related NPC^14,29–31^. However, in the current study, we only found that EBV positive patients had more chance to have lower CD8% and higher NK%, no significant association of EBV positive with other peripheral immune parameters in advanced NPC was found. Previous studies reported that EBV DNA titers correlated with tumor burden, remission, and recurrence and could precede tumor development of NPC^32–35^. Thus, we further analyzed the association of the concentration of plasma EBV DNA with immune parameters. Inconsistent with Williams’s study^36^, our data showed that patients with normal NK cell count had a higher EBV DNA load in peripheral blood. Williams et al.^36^ found that higher NK cell counts were significantly related to lower EBV DNA load in blood in infectious mononucleosis. This unexpected result might be explained by the fact that normal or higher amounts of NK cells killed more tumor cells and released more EBV DNA into the peripheral blood. However, this hypothesis needs to be validated in a further large size sample analysis.

Previous studies^7,37^ revealed that higher EBV DNA level was an unfavorable factor for PFS and OS in NPC patients without metastasis treated with radiation therapy and in locally advanced NPC patients treated with concurrent chemoradiotherapy. Consistent with these studies, our study also showed that EBV infection was a significant and independent worse predictor of PFS and OS in patients with advanced NPC, regardless of whatever therapy they may have received. The prognostic value of HBsAg status for advanced NPC patients is still controversial. The study of Liu et al.^24^, revealed that HBV infection was associated with poor prognosis in patients with locoregionally advanced NPC. However, Xu et al.^25^, failed to confirm that HBsAg ( +) was an independent predictor in patients with NPC. Similar to Xu’s reports^25^, we also did not found the significant prognostic value of HBsAg status in this cohort of patients.

There are some limitations to our study. First, this study was conducted retrospectively and the sample size was small. The results were not verified in a validation cohort. Second, the details of the diagnosis and treatment of newly diagnosed patients in the external hospital cannot be fully documented, and there was heterogeneity in the patient treatment before and after the diagnosis of IV stage, it was hard to analyze the effects of different treatment methods on the peripheral immune indicators. Third, this study did not include some comorbidity such as diabetes, rheumatic disease, and autoimmune disease to statistically analyze, which may also affect circulating immune cells.

In summary, EBV infection would raise the risk of liver metastasis and is a significant and independent unfavorable predictor of PFS and OS patients with advanced NPC. EBV positive patients had more chance to have lower CD8% and higher NK%, while the EBV viral load of patients with lower NK cell count was significantly associated with lower EBV viral load. However, it seems no relationship between the HBsAg status and liver metastasis and long-term survival in advanced NPC.

### Ethics statement

The study was approved by the Chongqing University Cancer Hospital Ethics Committee. All methods were carried out in accordance with the approved guidelines. For this was a retrospective analysis of routine data, we requested and were granted a waiver of individual informed consent from the ethics committee. Patient records/information was anonymized and de-identified prior to analysis.

### Data availability

All datasets generated for this study are included in the manuscript.

## Supplementary Information

Supplementary Information 1.
