# Supplementary material for: Association of Epstein-Barr virus infection with peripheral immune parameters and clinical outcome in advanced nasopharyngeal carcinoma
Source: Sci Rep. 2020 Dec 15;10:21976. doi: 10.1038/s41598-020-78892-0 (PMC7738521; doi:10.1038/s41598-020-78892-0)
Supplement: Supplementary file 1 — Supplementary Information 1. [file 41598_2020_78892_MOESM1_ESM.docx]

**Association of Epstein-Barr virus infection with peripheral immune parameters and clinical outcome in advanced nasopharyngeal carcinoma**

Dan Tao^1^, Ningning Zhang^2*^, Qingqing Huang^3^, Chuang Ge^4^, Qicheng Li^1^, Shujie Li^1^, Kegui Weng^1^, Qishuai Guo^1^, Jiangdong Sui^1^, Can Wang^1^, Xin Zhang^1^, Ying Wang^1*^

Supplementary Table 1.The relationship between clinicopathologic features and indexes of immunologic function

| Variables | CD3% | | P | CD3 | | P | CD8% | | P | CD8 | | P | CD4% | | P | CD4 | | P |
| --- | --- | --- | --- | --- | --- | --- | --- | --- | --- | --- | --- | --- | --- | --- | --- | --- | --- | --- |
|  | L | N |  | L | N |  | L | N |  | L | N |  | L | N |  | L | N |  |
| Age yrs  < 50  ≥ 50 | 10  10 | 63  63 | 1.000 | 52  58 | 21  15 | 0.249 | 2  5 | 71  68 | 0.245 | 46  49 | 27  24 | 0.603 | 23  36 | 50  37 | **0.028** | 54  59 | 19  14 | 0.322 |
| Sex  Male  Female | 18  2 | 95  31 | 0.245 | 81  29 | 32  4 | 0.058 | 6  1 | 107  32 | 0.590 | 73  22 | 40  11 | 0.827 | 46  13 | 67  20 | 0.892 | 82  31 | 31  2 | **0.010** |
| Smoker  Yes  No | 9  11 | 65  61 | 0.584 | 50  60 | 24  12 | **0.027** | 5  2 | 69  70 | 0.261 | 45  50 | 29  22 | 0.274 | 30  29 | 44  43 | 0.974 | 51  62 | 23  10 | **0.013** |
| T-stage  Tx-3  T4 | 15  5 | 90  36 | 0.741 | 82  28 | 23  13 | 0.217 | 6  1 | 99  40 | 0.688 | 70  25 | 35  16 | 0.517 | 45  14 | 60  27 | 0.335 | 83  30 | 22  11 | 0.445 |
| N-stage  N0-2  N3 | 16  4 | 86  40 | 0.288 | 81  29 | 21  15 | 0.082 | 3  4 | 99  40 | 0.199 | 73  22 | 29  22 | **0.012** | 48  11 | 54  33 | **0.013** | 82  31 | 20  13 | 0.188 |
| M-stage  M0  M1 | 2  18 | 38  88 | 0.060 | 24  86 | 16  20 | **0.008** | 1  6 | 33  100 | 0.717 | 19  76 | 21  30 | **0.006** | 12  47 | 28  59 | 0.115 | 27  86 | 13  20 | 0.079 |
| Clinical stage  IVa  IVb  IVc | 2  0  18 | 16  22  88 | 0.102 | 10  14  86 | 8  8  20 | **0.033** | 1  0  6 | 17  22  100 | 0.309 | 9  10  76 | 9  12  30 | **0.023** | 6  6  47 | 12  16  59 | 0.268 | 12  15  86 | 6  7  20 | 0.229 |
| Lung M  Yes  No | 4  16 | 28  98 | 0.823 | 25  85 | 7  29 | 0.679 | 3  4 | 29  110 | 0.170 | 22  73 | 10  41 | 0.621 | 9  50 | 23  64 | 0.109 | 24  89 | 8  25 | 0.714 |
| Liver M  Yes  No | 3  17 | 35  91 | 0.226 | 30  80 | 8  28 | 0.549 | 1  6 | 37  102 | 0.776 | 28  67 | 10  41 | 0.195 | 15  44 | 23  64 | 0.891 | 31  82 | 7  26 | 0.474 |
| Bone M  Yes  No | 13  7 | 40  86 | **0.004** | 48  62 | 5  31 | **0.001** | 4  3 | 49  90 | 0.256 | 42  53 | 11  40 | **0.007** | 28  31 | 25  62 | **0.021** | 48  65 | 5  28 | **0.004** |
| Prior R  Yes  No | 14  6 | 45  81 | **0.004** | 55  55 | 4  32 | **0.000** | 2  5 | 57  82 | 0.702 | 49  46 | 10  41 | **0.000** | 38  21 | 21  66 | **0.000** | 55  58 | 4  29 | **0.000** |
| Prior C  Yes  No | 14  6 | 44  82 | **0.003** | 56  54 | 2  34 | **0.000** | 2  5 | 56  83 | 0.703 | 49  46 | 9  42 | **0.000** | 37  22 | 21  66 | **0.000** | 54  59 | 4  29 | **0.000** |
| EBV status  Positive  Negative | 13  7 | 74  52 | 0.596 | 66  44 | 21  15 | 0.860 | 7  0 | 80  59 | **0.042** | 60  35 | 27  24 | 0.230 | 31  28 | 56  31 | 0.153 | 69  44 | 18  15 | 0.502 |
| Variables | CD4/CD8 | | P | NK% | | P | NK | | P | BC% | | P | BC | | P | CD45 | | P |
|  | L | N |  | L | N |  | L | N |  | L | N |  | L | N |  | L | N |  |
| Age yrs  < 50  ≥ 50 | 17  24 | 56  49 | 0.197 | 3  3 | 70  70 | 1.000 | 26  21 | 47  52 | 0.376 | 11  20 | 62  53 | 0.069 | 27  43 | 46  30 | **0.008** | 58  59 | 15  14 | 0.836 |
| Sex  Male  Female | 30  11 | 83  22 | 0.445 | 4  2 | 109  31 | 0.618 | 32  15 | 81  18 | 0.064 | 23  8 | 90  25 | 0.631 | 54  16 | 59  17 | 0.944 | 87  30 | 26  3 | 0.078 |
| Smoker  Yes  No | 21  20 | 53  52 | 0.936 | 3  3 | 71  69 | 0.973 | 24  23 | 50  49 | 0.950 | 14  17 | 60  55 | 0.488 | 34  36 | 40  36 | 0.624 | 54  63 | 20  9 | **0.028** |
| T-stage  Tx-3  T4 | 33  8 | 72  33 | 0.150 | 6  0 | 99  41 | 0.185 | 35  12 | 70  29 | 0.637 | 23  8 | 82  33 | 0.751 | 52  18 | 53  23 | 0.541 | 87  30 | 18  11 | 0.187 |
| N-stage  N0-2  N3 | 33  8 | 69  36 | 0.080 | 1  5 | 101  39 | **0.010** | 30  17 | 72  27 | 0.274 | 23  8 | 79  36 | 0.554 | 51  19 | 51  25 | 0.449 | 85  32 | 17  12 | 0.141 |
| M-stage  M0  M1 | 8  33 | 32  73 | 0.182 | 4  2 | 36  104 | **0.048** | 13  34 | 27  72 | 0.961 | 11  20 | 29  86 | 0.255 | 20  50 | 20  56 | 0.760 | 27  90 | 13  16 | **0.019** |
| Clinical stage  IVa  IVb  IVc | 4  4  33 | 14  18  73 | 0.394 | 0  4  2 | 18  18  104 | **0.009** | 3  10  34 | 15  12  72 | 0.153 | 5  6  20 | 13  16  86 | 0.536 | 7  13  50 | 11  9  56 | 0.425 | 11  16  90 | 7  6  16 | 0.056 |
| Lung M  Yes  No | 5  36 | 27  78 | 0.076 | 6  0 | 32  108 | 0.185 | 9  38 | 23  76 | 0.577 | 4  27 | 28  87 | 0.172 | 19  51 | 13  63 | 0.143 | 28  89 | 4  25 | 0.237 |
| Liver M  Yes  No | 13  28 | 25  80 | 0.328 | 0  6 | 38  102 | 0.313 | 12  35 | 26  73 | 0.925 | 6  25 | 32  83 | 0.340 | 14  56 | 24  52 | 0.111 | 31  86 | 7  22 | 0.796 |
| Bone M  Yes  No | 20  21 | 33  72 | **0.050** | 1  5 | 52  88 | 0.417 | 17  30 | 36  63 | 0.982 | 11  20 | 42  73 | 0.915 | 27  43 | 26  50 | 0.584 | 47  70 | 6  23 | 0.051 |
| Prior R  Yes  No | 28  13 | 31  74 | **0.000** | 0  6 | 59  81 | 0.081 | 20  27 | 39  60 | 0.716 | 14  17 | 45  70 | 0.544 | 32  38 | 27  49 | 0.210 | 55  62 | 4  25 | **0.001** |
| Prior C  Yes  No | 26  15 | 32  73 | **0.000** | 0  6 | 58  82 | 0.081 | 21  26 | 37  62 | 0.399 | 13  18 | 45  70 | 0.777 | 31  39 | 27  49 | 0.280 | 55  62 | 3  26 | **0.000** |
| EBV status  Positive  Negative | 21  20 | 66  39 | 0.198 | 1  5 | 86  54 | **0.040** | 24  23 | 63  36 | 0.148 | 17  14 | 70  45 | 0.544 | 45  25 | 42  34 | 0.267 | 69  48 | 18  11 | 0.761 |
